# Sperm protein 17 is expressed in human nervous system tumours

**DOI:** 10.1186/1471-2407-6-23

**Published:** 2006-01-26

**Authors:** Fabio Grizzi, Paolo Gaetani, Barbara Franceschini, Antonio Di Ieva, Piergiuseppe Colombo, Giorgia Ceva-Grimaldi, Angelo Bollati, Eldo E Frezza, E Cobos, Riccardo Rodriguez y Baena, Nicola Dioguardi, Maurizio Chiriva-Internati

**Affiliations:** 1 Scientific Direction, Istituto Clinico Humanitas, IRCCS, 20089 Rozzano, Milan, Italy; 2 Department of Neurosurgery, Istituto Clinico Humanitas, IRCCS, 20089 Rozzano, Milan, Italy; 3 Department of Pathology, Istituto Clinico Humanitas, IRCCS, 20089 Rozzano, Milan, Italy; 4 "Michele Rodriguez" Foundation. Scientific Institute for Quantitative Measures in Medicine, 20100 Milan, Italy; 5 Department of Neurosurgery, University of Brescia, Spedali Civili, Italy; 6 Department of Surgery, Texas Tech University Health Sciences Center and Southwest Cancer Treatment and Research Center, 79430 Lubbock, Texas, USA; 7 Department of Internal Medicine, Texas Tech University Health Sciences Center and Southwest Cancer Treatment and Research Center, 79430 Lubbock, Texas, USA; 8 Department of Microbiology and Immunology, Texas Tech University Health Sciences Center and Southwest Cancer Treatment and Research Center, 79430 Lubbock, Texas, USA

## Abstract

**Background:**

Human sperm protein 17 (Sp17) is a highly conserved protein that was originally isolated from a rabbit epididymal sperm membrane and testis membrane pellet. It has recently been included in the cancer/testis (CT) antigen family, and shown to be expressed in multiple myeloma and ovarian cancer. We investigated its immunolocalisation in specimens of nervous system (NS) malignancies, in order to establish its usefulness as a target for tumour-vaccine strategies.

**Methods:**

The expression of Sp17 was assessed by means of a standardised immunohistochemical procedure [(mAb/antigen) MF1/Sp17] in formalin-fixed and paraffin embedded surgical specimens of NS malignancies, including 28 neuroectodermal primary tumours (6 astrocytomas, 16 glioblastoma multiforme, 5 oligodendrogliomas, and 1 ependymoma), 25 meningeal tumours, and five peripheral nerve sheath tumours (4 schwannomas, and 1 neurofibroma),.

**Results:**

A number of neuroectodermal (21%) and meningeal tumours (4%) were found heterogeneously immunopositive for Sp17. None of the peripheral nerve sheath tumours was immunopositive for Sp17. The expression pattern was heterogeneous in all of the positive samples, and did not correlate with the degree of malignancy.

**Conclusion:**

The frequency of expression and non-uniform cell distribution of Sp17 suggest that it cannot be used as a unique immunotherapeutic target in NS cancer. However, our results do show the immunolocalisation of Sp17 in a proportion of NS tumour cells, but not in their non-pathological counterparts. The emerging complex function of Sp17 makes further studies necessary to clarify the link between it and immunopositive cells.

## Background

Brain and other nervous system (NS) tumours are a group of neoplasms that vary in terms of their site of origin, morphological features, genetic alterations, growth potential, invasiveness, tendency to progression and recurrence, and response to treatments [1-4]. Their still highly unfavourable prognosis underlines the need for new therapeutic approaches, one of the most attractive of which is targeted immunotherapy (any approach aimed at mobilising or manipulating a patient's immune system to treat or cure disease), which is widely considered to be more specific and less toxic than conventional surgery, chemotherapy or radiation therapy [5,6]. However, although the blood-brain barrier and the claimed immunological privilege of the brain are not necessarily obstacles to effective brain immunotherapy, these strategies are currently limited by the paucity of cloned NS target antigens and the fact that our understanding of anti-tumour immune responses in the NS is frequently extrapolated from other tissues having little in common with it [7,8].

A cancer-testis (CT) family of tumour-associated antigens has been identified and their encoding genes extensively investigated [9-13]. Their immunogenicity and restricted expression in normal tissues makes them ideal molecules for immunotherapeutic procedures [9,10].

Sperm protein 17 (Sp17) has recently been entitled as novel CT in ovarian cancer, multiple myeloma and other blood malignancies [14-16], and mRNA encoding Sp17 has been found in two myeloma cell lines, 17% of a series of patients with multiple myeloma, and in primary tumour cells from 70% of a series of patients with ovarian carcinoma [14,15]. At protein level, Sp17 has been found in human germinal cells of the testis other than spermatogonia [17], as well as in the ciliated epithelia of the respiratory airways and both male and female reproductive systems [18]. It has also recently been identified in the synoviocytes of females with rheumatoid arthritis [19], and in the melanophages of cutaneous melanocytic lesions [20]. Although these findings have all raised the question as to whether Sp17 may be a useful target for tumour immunotherapy [21-23], they also highlight the fact that its tissue distribution in humans is more complex than originally thought.

The expression frequencies of many CT antigens have been determined in a variety of neoplasms [11], but little is known about their expression in human NS tumours.

The aim of this immunohistochemical study was to investigate the frequency of Sp17 expression at protein level and its cell pattern distribution in various histological subtypes of human NS tumours, including neuroectodermal, meningeal and nerve sheath lesions.

## Methods

### Patients

The study was conducted in accordance with the guidelines of the Ethics Committee of the hospital treating the patients (Istituto Clinico Humanitas, Rozzano, Milan, Italy), all of whom were fully informed of the possible discomfort and risks of surgical treatment.

All of the analysed tissues were taken from patients admitted to the Istituto Clinico Humanitas, Rozzano, Milan, between 2002 and 2004. The patients' mean age was 58 years (range: 18–79), and the group consisted of 26 males and 32 females.

### Tissue specimens

The NS tumour specimens represented 28 neuroectodermal tumours (6 astrocytomas, 16 glioblastomas, 5 oligodendrogliomas, and 1 ependymoma), 25 meningeal tumours, and five nerve sheath tumours (4 schwannomas, 1 neurofibroma).

To evaluate whether Sp17 is expressed at protein level in non-pathological NS tissue, three further specimens were taken from one male and two female patients (mean age 58 ± 15 years) who underwent surgical resection for neoplastic disease and were sampled at the periphery of the lesions. The absence of cell or tissue pathologies was subsequently confirmed by microscopy.

All of the specimens were fixed in 10% neutral buffered formalin (~12 hours) and embedded in paraffin.

### Reverse transcription-polymerase chain reaction (RT-PCR) amplification of Sp17 mRNA

A panel of normal tissue total RNA was obtained from Strategene Corp. (La Jolla, CA). All of the RNAs were first treated with DNAse I (Ambion, Austin, TX) to remove genomic DNA contamination, and first- strand cDNA was synthesised from 1 μg by means of random hexamer primers. The primers for 5' Sp17 PCR was 5'-GGCAGTTCTTACCAAGAAGAT-3', and that for 3' Sp17 PCR was 5'- GGAGGTAAAACCAGTGTCCTC-3', both of which amplify a complementary DNA (cDNA) of approximately 500 base-pairs. PCR was performed by means of 35 amplification cycles at an annealing temperature of 55°C. The positive control amplification contained a plasmid with the Sp17 cDNA and a negative control of the PCR reaction mixture except for the substitution of cDNA by water. RNA integrity in each sample was checked by amplifying a β-actin gene segment. The successful removal of DNA contamination was confirmed by amplifying the RNA in each sample without the reverse transcription reaction. The PCR products were visualised on an ethidium bromide agarose gel for a DNA band of the expected size. All of the results were confirmed by two independent RT-PCRs.

### Immunohistochemistry

Two-μm thick sections were cut and processed for immunohistochemistry. After deparaffining and rehydration, the sections were immersed in a bath (Dako, Milan, Italy) for antigen retrieval for 45 min at 98°C in a freshly made EDTA 1 mM solution, incubated with 3% H_2_O_2 _for 15 min, and then treated with primary antibodies raised against Sp17 [monoclonal murine anti-Sp17 (Sp17MF1), dilution 1:100 in phosphate buffered saline, PBS] at room temperature for two hours, or with 1 mg /ml mouse IgG1 (Dako, Milan, Italy) as a negative control. This was followed by 30 min incubation with the DAKO Envision system (Dako, Milan, Italy). 3,3'-diaminobenzidine tetrahydrochloride (Sigma Ltd, Missouri, USA) was used as a chromogen to yield brown reaction products.

The testis specificity of the binding of the Sp17 antibodies has been confirmed in competitive binding assays showing the abrogation of testicular staining if the Sp17 antibodies are pre-incubated with a very high concentration (100 μg/ml) of soluble Sp17 recombinant protein [24]. The specificity of the primary antibodies raised against Sp17 was also shown by Western blotting analyses [17,18,25].

The nuclei were lightly counterstained with hematoxylin solution (Medite, Bergamo, Italy).

The immunohistochemical reaction was observed using a light microscope (Leica DMLA, Italy) at 40× and 100× objective magnifications, and was semi-quantitatively graded into four classes by two expert neuropathologists on the basis of the frequency of Sp17 in NS cancer cells: negative = no immunopositive cells (*x*); + = low frequency (*x *≤ 25%); ++ = moderate frequency (25% <*x *≤ 50%); +++ = high frequency (50% <*x *≤ 75%); ++++ = very high frequency (75% <*x *≤ 100%).

### Statistical analysis

All of the data are expressed as mean values ± standard deviation. The results were analysed using Statistica software (StatSoft Inc., Tulsa, OK, USA). Univariate analysis was performed by means of the Student *t *or chi-square test as required for parametric and categorical variables. A *p *value of less than 0.05 was considered to be statistically significant.

## Results

### Patient baseline characteristics

Table 1 shows the investigated NS lesions and the patients' baseline characteristics. Despite the limited number of cases, the mean age of the patients with oligodendrogliomas (45 ± 10 years) was significantly different from that of those with meningiomas (60 ± 14 years; p = 0.03) or schwannomas (65 ± 9 years; p = 0.01). There was no significance difference in gender distribution between the neoplastic histological subtypes.

### RT-PCR amplification of Sp17 mRNA

**Figure 1:**
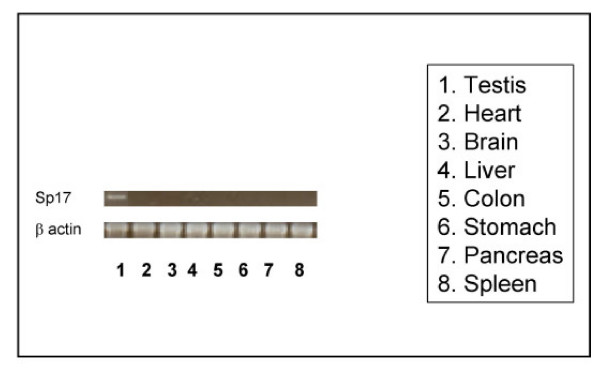
RT-PCR showing Sp17 transcripts in testis, but not in brain, colon, heart, liver, stomach, pancreas, and spleen (a). Control amplification for the β-actin gene segment (b).

Using a pair of sequence-specific primers, mRNA encoding Sp17 was found in testis, but not in brain, colon, heart, liver, stomach, pancreas, and spleen (Fig. 1).

### Immunohistochemistry

**Figure 2:**
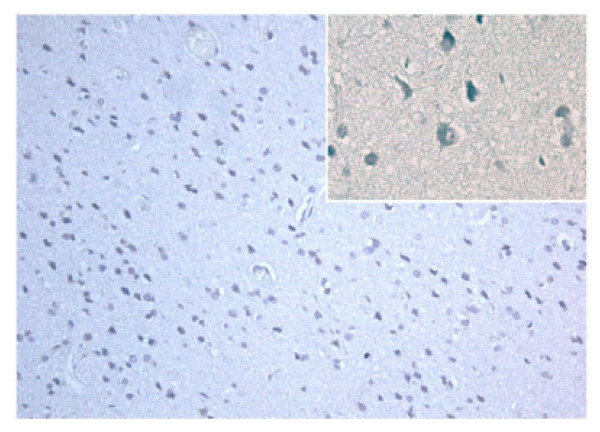
Immunohistochemistry of Sp17 in non-pathological nervous system tissues. There were no immunopositive cells (original magnification, 20×, 40× inset).

**Figure 3:**
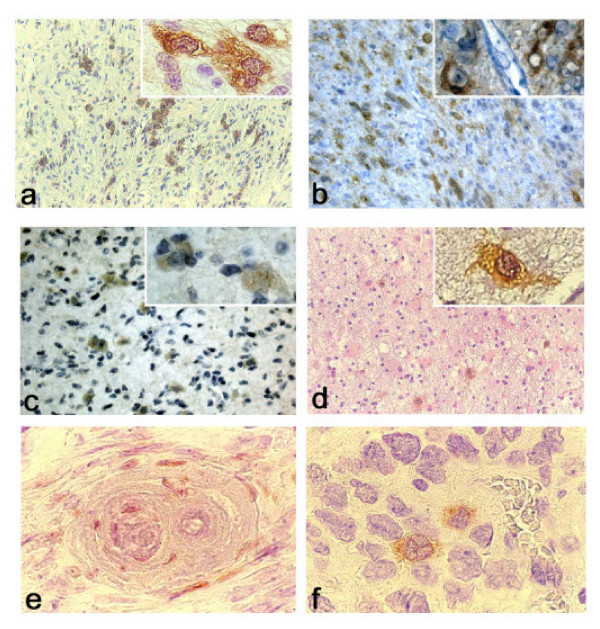
Immunohistochemistry of Sp17 in nervous system neoplasms: *a*-*c*. glioblastomas; *d*. astrocytoma; *e*. meningioma; *f*. ependimoma. In all cases, Sp 17 was localised in the cytoplasm of a few isolated and scattered tumoral cells. (*a*-*d*, 40× original magnification, 100× insets; *e*-*f*, 100× original magnification).

Table 2 shows the frequencies of Sp17 expression at protein level. All three non-pathological NS specimens were immunonegative (Fig. 2), but Sp17 was found in 21% of the neuroectodermal and 4% of the meningeal lesions. Excluding the only ependymoma (which was immunopositive), the highest frequency was among the astrocytomas. None of the nerve sheath tumours was immunopositive. In all of the immunopositive lesions, Sp17 was localised in the cytoplasm of a subset of cancer cells. Each immunopositive case was semi-quantitatively graded by two expert neuropathologists (Tab. 3). The expression pattern was always heterogeneous (Fig. 3) and did not correlate with the degree of malignancy.

Sp17 was expressed in six female patients, who accounted for 86% of the immunopositive patients and one male. Considering the presumed cell of origin of the lesions, Sp17 was found in two females (100%) and no males with astrocytoma; two females (18%) and one male (20%) with glioblastoma; one female (5.8%) and no males with meningioma; and one female (100%) with ependymoma.

There was no difference in the mean age of the patients who were Sp17 immunopositive or immunonegative.

## Discussion

Despite advances in surgical techniques, imaging methodologies and adjuvant radiation therapy, the unfavourable prognosis of most brain and other NS tumours [1-3] has prompted an intensive search for effective treatment alternatives. Novel immunostimulatory strategies, such as immunogenic therapy, directed cytokine delivery and dendritic cell manipulation, have recently been used in patients with malignant NS tumours in an attempt to improve the results obtained with surgery, chemotherapy and radiotherapy [7,8].

Since the first cloning of a human tumour antigen, melanoma antigen-1 (MAGE-1), the identification of tumour antigens capable of inducing an immune response in cancer patients, and the development of vaccines targeting these antigens, have been formidable tasks confronting tumour immunologists [5,6,9-13]. It has long been accepted that immunotherapy is a powerful cancer treatment, but it has been mainly limited by a poor understanding of the complexity of human beings, the immune system, and highly intricate host-tumour interactions [23,26].

The introduction of T-cell epitope cloning and, subsequently, a method based on the serological analysis of recombinant tumour cDNA expression libraries using autologous serum (SEREX), has led to the identification of almost 44 CT gene families [10,11]. Sp17 has recently been included among them because, although it was originally thought to be gamete-specific, it has also been identified in various human somatic and tumoral tissues [14-16,18-20,27-29]. The expression frequencies of many CT antigens have been determined in many neoplasms, but little is known about their expression in human NS tumours [11].

We show that Sp17 is heterogeneously expressed at protein level in a proportion of neuroectodermal and meningeal tumours, but not in nerve sheath neoplasms or non-pathological NS tissues.

Sahin *et al. *(2000) have recently investigated the expression of seven CT genes (MAGE-3, NY-ESO-1, HOM-MEL-40/SSX-2, SSX-1, SSX-4, HOM-TES-14/SCP-1, and HOM-TES-85) in a series of human brain tumour specimens, and concluded that a majority of oligoastrocytomas and astrocytomas might be amenable to specific immunotherapeutic interventions, although the identification of additional tumour-specific antigens frequently expressed in gliomas should allow for the development of widely applicable polyvalent glioma vaccines [30].

However, it must be pointed out that the authors investigated mRNA expression, which does not necessarily correlate directly with protein expression. The discrepancies between the frequencies of gene and protein expression may reflect variations in tissue sampling, the heterogeneity tumour cells, or differences in the sensitivity of detection [10,11,26,31]. The expression of CT antigens in clinical material has mainly been studied by RT-PCR at gene expression level, but this approach is partially limited by the fact that it does not allow the quantification of cancer cells positive for CT antigens [32]. On the contrary, the availability of specific antibodies enables the recognition of candidate antigens in examined tissues, thus highlighting both the number and type of cells that are immunopositive for a given antigen [11,23,32]; however, the number of monoclonal antibodies against CT proteins that can be used in immunohistochemical or biochemical analyses of protein expression is still relatively limited [11,32].

On the basis of our findings, it can be said that:

*a*. Sp17 can be recognised at protein level in various neuroectodermal and meningeal tumours, but not in nerve sheath tumours.

*b*. Sp17 is localised in the cytoplasm of a variable number of tumoral cells (Fig. 3). The focal expression of Sp17 in NS cancer cells suggests that this antigen might not be sufficient as a unique target for immunotherapeutic approaches. It is now widely accepted that a prerequisite for the success of any tumour-specific therapeutic strategy is the existence and identification of antigen targets that are not only exclusively or preferentially expressed in malignant tissues, but also expressed in a high percentage of neoplastic cells in a large number of patients [10-12,23,31,33].

*c*. Although the clinical and patient characteristics of our study population were similar to those described in other series, its redistribution on the basis of immunohistochemical findings highlights the fact that 86% of the samples immunopositive for Sp17 were taken from female patients. Although this finding needs to be confirmed in a large cohort of patients, it extends those of a previous study by Takeoka *et al. *(2000), who found Sp17 expressed in the synoviocytes of females with rheumatoid arthritis [19].

The variable frequency of the expression of CT antigens, and their uneven distribution in cancer cells [34], have been recently been attributed to the spatial and temporal complexity of tumoral lesions and their non-linear interactions with the body environment [26,35-37]. These basic characteristics have led to the development of a system-level approach that is not only intended to generate a human cancer map of the expression of candidate antigens, but also to highlight common features underlying tumours of unrelated histological origin, thus improving our basic knowledge of tumour complexity [21].

Our study is the first to show the expression of Sp17 at protein level in a proportion of NS tumours but not in non-pathological NS tissues. This extends our knowledge of CT antigens and NS cancer [38-41], and reinforces the need for further studies aimed at clarifying the complex function of Sp17, which clearly seems to go beyond the simple role in the fertilisation process that was originally attributed to it.

## Competing interests

The author(s) declare that they have no competing interests.

## Authors' contributions

FG, MCI conceived, coordinated and designed the study, and drafted the manuscript; PG, BF, ADI, PC, GCG, AB, EEF, EC, RRB, ND participated in designing the study and drafting the manuscript. All of the authors have read and approved the final manuscript.

## Pre-publication history

The pre-publication history for this paper can be accessed here:



## Figures and Tables

**Table 1:** Patient baseline characteristics.

**Tumour histological subtype**	**Patients (n)**	Age* (years)	**Sex**
Astrocytoma	6	60 ± 20	4M/2F
Oligodendroglioma	5	45 ± 10	5M
Ependymoma	1	22	1F
Glioblastoma	16	59 ± 17	5M/11F
Meningioma	25	60 ± 14	8M/17F
Swhanoma	4	65 ± 9	3M/1F
Neurofibroma	1	32	1M

*Data are expressed as mean ± standard deviation

**Table 2:** Sperm protein 17 expression frequencies in different histological subtypes of primary nervous system neoplasms. The lesions are categorised on the basis of their presumed cell of origin.

**Cell of origin**	**number positive/total number tested**
***Glial cell***	
Astrocytoma	2/6
Oligodendroglyoma	0/5
Ependymoma	1/1
Glioblastoma	3/16

***Arachinodal cell***	
Meningioma	1/25

***Nerve Sheath cell***	
Swhanoma	0/4
Neurofibroma	0/1

**Table 3:** Semi-quantitative grading of Sp17 expression in NS tumours.

**Sample**	**Immunohistochemistry**	**Pattern**
Astrocytoma 1	+++	Cyto
Astrocytoma 2	+++	Cyto
Ependymoma	++	Cyto
Glioblastoma 1	++	Cyto
Glioblastoma 2	+++	Cyto
Glioblastoma 3	+++	Cyto
Meningioma	+	Cyto

**Notes**: + = low frequency (x ≤ 25%) of cells immunopositive for Sp17; ++ = moderate frequency (25% < x ≤ 50%) of cells immunopositive for Sp17; +++ = high frequency (50% < x ≤ 75%) of cells immunopositive for Sp17; ++++ = very high frequency of cells immunopositive for Sp17 (75% < x ≤ 100%). Cyto = cytoplasmic Sp17 localization.
